# The Great Acceleration of fragrances and PAHs archived in an ice core from Elbrus, Caucasus

**DOI:** 10.1038/s41598-020-67642-x

**Published:** 2020-06-30

**Authors:** Marco Vecchiato, Andrea Gambaro, Natalie M. Kehrwald, Patrick Ginot, Stanislav Kutuzov, Vladimir Mikhalenko, Carlo Barbante

**Affiliations:** 1 Institute of Polar Sciences (ISP-CNR), Via Torino 155, Venezia-Mestre, 30172 Venice, Italy; 2 Department of Environmental Sciences, Informatics and Statistics (DAIS), Ca’ Foscari University of Venice, Via Torino 155, Venezia-Mestre, 30172 Venice, Italy; 3 Geosciences and Environmental Change Science Center, U.S. Geological Survey, MS 980, Denver, CO 80225 USA; 4 Université Grenoble Alpes, IRD, CNRS, G-INP, Institut Des Géosciences de L’Environnement (IGE), 38402 Grenoble, France; 5 Institute of Geography, Russian Academy of Sciences, Moscow, Russia 119017

**Keywords:** Cryospheric science, Geochemistry

## Abstract

The Great Acceleration of the anthropogenic impact on the Earth system is marked by the ubiquitous distribution of anthropogenic materials throughout the global environment, including technofossils, radionuclides and the exponential increases of methane and carbon dioxide concentrations. However, personal care products as direct tracers of human domestic habits are often overlooked. Here, we present the first research combining fragrances, as novel personal care products, and polycyclic aromatic hydrocarbons (PAHs) as combustion and industrial markers, across the onset of the Great Acceleration in the Elbrus, Caucasus, ice core. This archive extends from the 1930s to 2005, spanning the profound changes in the relationship between humans and the environment during the twentieth century. Concentrations of both fragrances and PAHs rose throughout the considered period, reflecting the development of the Anthropocene. However, within this rising trend, remarkable decreases of the tracers track the major socioeconomic crises that occurred in Eastern Europe during the second half of the twentieth century.

## Introduction

The recent influence of human activity on the global environment is so profound that a new geological epoch, the Anthropocene, was proposed. From the mid-twentieth century onwards the exponential growth in human population, economic activity and resource consumption created a global-scale change in the human signal, defined as the Great Acceleration^1,2^. Geochemical signatures due to novel anthropogenic materials, including new minerals, plastics and organic contaminants, can be detected in nearly every terrestrial environment. The cryosphere is particularly good at archiving this human signal, by preserving the deposited aerosols and chemicals in the ice^3,4^.

A variety of indicators related to human population growth demonstrate a marked increasing trend since the 1950s^5^, but few studies consider the evolution of personal care products as socioeconomic development tracers during the Great Acceleration^6^. The concentrations of these compounds in environmental matrices may be related to population size, changing industrial production and domestic behavior, as well as due to transport mechanisms^7^. The knowledge regarding the distribution of personal care products in the cryosphere is limited^8–10^ and no studies currently consider these contaminants in ice cores.

Compared to personal care products, more information is available for the presence of Polycyclic Aromatic Hydrocarbons (PAHs) in ice core archives. However, the related literature is restricted to a handful of studies, representing different Arctic^11,12^, Antarctic^13,14^ and high altitude environments^15–17^. PAHs are produced by the incomplete combustion of organic material and partially derive from natural sources, but mostly derive from anthropogenic emissions^18^. While this human signal can be detected in remote environments^19^, the majority of the research in mountain environments is from other Eurasian mountain ranges such as the Himalaya or the Alps, with only a few studies from the Caucasus.

**Figure 1:**
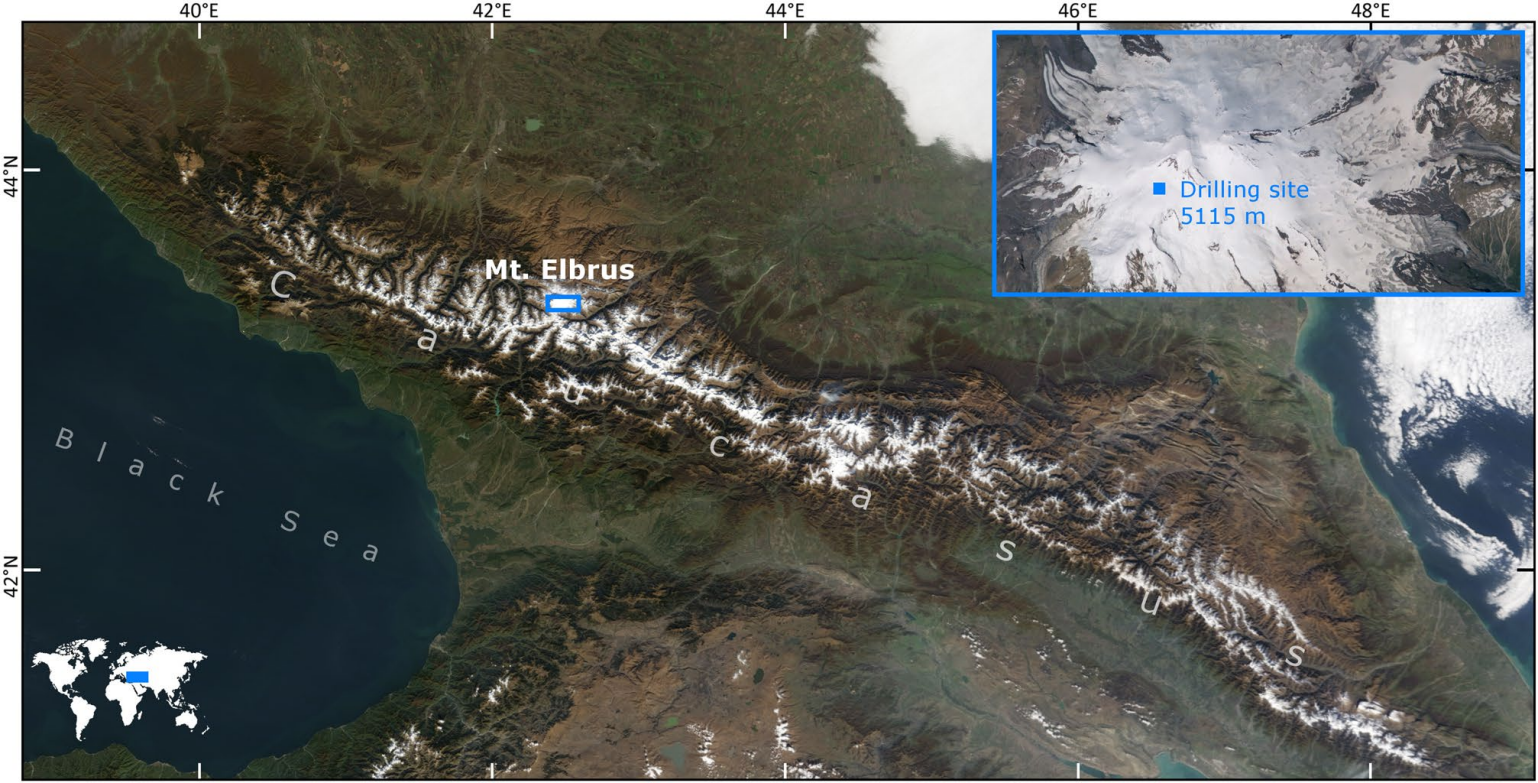
Location of the drill site on the western plateau of Elbrus, Caucasus. Adapted from visibleearth.nasa.gov (NASA, MODIS Terra, November 9, 2008).

Elbrus, the highest mountain in the Caucasus (5642 m a.s.l.), is an active volcano located between the Black and Caspian Seas (Fig. 1)^20^. The site lies on the border between subtropical and temperate climatic zones and is influenced by prevailing westerly winds, with air masses primarily originating from the Mediterranean and Eastern Europe^21,22^. However, specific meteorological events can transport dust from the Sahara and the Middle East to the surface of Elbrus^23–25^. A density plot of annual 10-day back-trajectories was computed for every 6 h for the period 1948–2013 using the NOAA-HYSPLIT model and NCEP/NCAR Reanalysis database (Figure S1)^26^ and demonstrates the provenance of air masses that reach the drill site.

In 2009 an ice core was drilled to bedrock on the western plateau of Elbrus (43° 20′53.9″ N, 42° 25′ 36.0″ E; 5,115 m a.s.l.), in a site with a high mean accumulation rate (1,455 mm w.e. year^−1^) and limited seasonal melting. This core represents one of the first high-resolution paleoclimate records in the region^20^. The annual mean air temperature at the drill site is approximately − 19 °C and the borehole temperature ranges from − 17 to − 12 °C at depths between 10 and 100 m. The firn-ice transition occurs at ~ 55 m^20^. The ice core has been extensively analyzed for major ions^20^, stable isotopes^21^ and black carbon^22^. Dust^25^ and sulphate^27^ concentrations substantially increase over the past few decades from their pre-industrial levels, where this rise is attributed to anthropogenic activity.

This study aims to decipher the anthropogenic signal of the Great Acceleration in the Elbrus ice core by analyzing two classes of organic contaminants: (1) PAHs as combustion and industrial activity products, and (2) personal care products as reflecting the evolution of human domestic habits. Most compounds of the latter category, such as cosmetics, toiletries or pharmaceuticals, have a low environmental mobility and are expected to be found close to their primary sources^9^. However, sufficiently volatile compounds may be transported to remote environments^8^. Long‐lasting and stable fragrances were recently detected in the coastal seawater of the Ross Sea, Antarctica^28^ and in seawater and snow samples from the settlement of Ny-Ålesund, Svalbard^8^. While Arctic and Antarctic research bases are local sources of fragrances, their presence in background clean areas may be due to long-range atmospheric transport (LRAT)^8^. A similar situation occurs in the Venice Lagoon, where sewage emits these contaminants into urban canals^29^, while marine currents and/or atmospheric deposition may transport fragrances to the open Mediterranean^30^.

The 17 studied fragrance compounds were initially selected due to features promoting their possible persistence in the environment. These properties include sufficient chemical stability to allow their application in aggressive commercial products, such as bleach and acid-based cleaners, as well as their perfumes that persist for a few weeks to months^29^. As opposed to other readily biodegradable or non-volatile personal care products, these compounds may be more easily transported to remote areas. Relevant physico-chemical information for the detected fragrances is included in Table SI1. The most widespread components in the above studies are allergenic and estrogenic Salicylate compounds. Their abundance may be due to their global use, where the low production prices of Benzyl, Hexyl and Amyl Salicylates (less than $5/kg) caused a rapid growth in worldwide consumption thereby meeting the High Production Volume (HPV) chemical criteria (> 5,000 tons/year)^31,32^.

This research represents the first investigation of personal care products in ice cores, as well as the first record of PAHs in the Caucasus. As PAHs are well-known environmental tracers, they provide trends against which to compare the evolution in fragrance concentrations and the changes in personal care product use.

## Results

**Figure 2:**
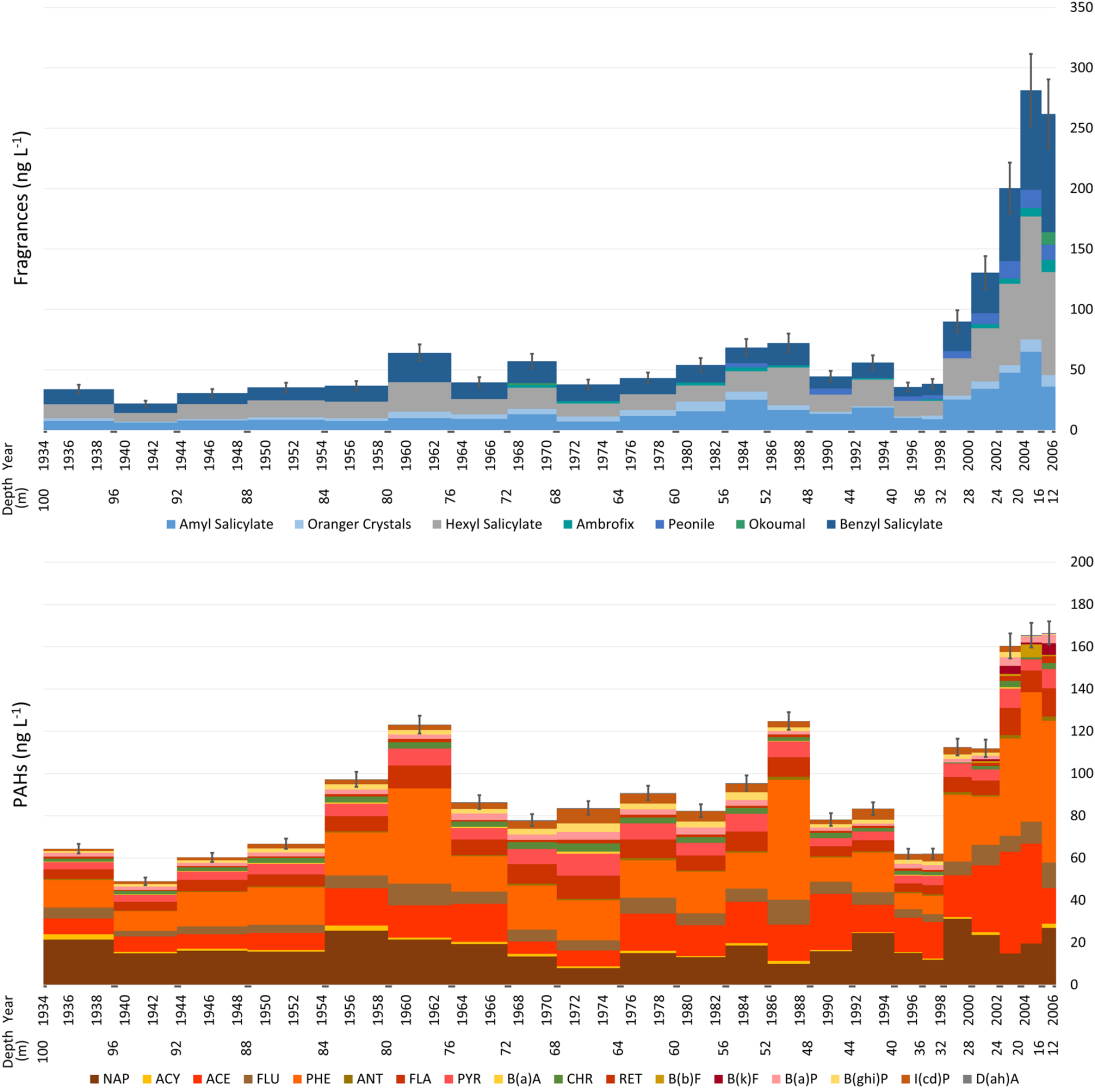
Fragrances and PAHs (ng L^−1^) in the Elbrus ice core with the year of snow deposition (CE) and the sample depth (m) reported along the abscissas. Error bars correspond to the standard deviation percentage of the method precision. Seasonal dating uncertainties of the analyzed ice core sections (0.5 years) are shown below the x-axes. Only detected compounds are displayed. Note the different y-axis scales for the fragrances and PAHs.

Concentrations of fragrances increase throughout the studied period (Fig. 2). In samples corresponding to the 1930s and 1940s, total concentrations were between 20 and 30 ng L^−1^ and continued increasing until peaking at 281 ng L^−1^ in 2004. The corresponding flux estimates demonstrate a 20-fold increase in the total deposition of fragrances, from ≈ 20 µg m^−2^ year^−1^ in the deepest layers, to 565 µg m^−2^ year^−1^ in the most recent years.

Single compounds demonstrate similar trends to the total fragrances. The three most abundant single compounds throughout the entire timespan are Amyl Salicylate, Hexyl Salicylate and Benzyl Salicylate, constituting 80–96% of the total fragrance concentrations in the samples (Table SI3) which is similar to previous studies^8,28–30^. Relative contributions of Amyl, Hexyl and Benzyl Salicylate vary only slightly through time (Fig. 2), respectively representing 25 ± 5%, 34 ± 5% and 36 ± 6% of the total sample fragrance concentrations.

In addition to the to the Salicylates, Oranger Crystals (2-acetonaphthone) is the only other fragrance detected in every sample (0.8–10 ng L^−1^). The Oranger Crystals flux increases from ≈ 1 µg m^−2^ year^−1^ during the 1930s and 1940s, to 21 µg m^−2^ year^−1^ at the top of the core. Ambrofix (Dodecahydro-3a,6,6,9a-tetramethylnaphtho[2,1-b]furan) and Peonile (2-cyclohexylidene-2-phenylacetonitrile) were only detected in the upper samples, respectively appearing at the end of the 1960s and in approximatively 1983/84 (Fig. 2) with a gradual increase in their concentrations after their initial appearance. Okoumal (2,4-Dimethyl-2-(1,1,4,4-tetramethyltetralin-6-yl)-1,3-dioxolane) is only occasionally observed in the uppermost layers as well as in the 1968–1970 sample.

**Figure 3:**
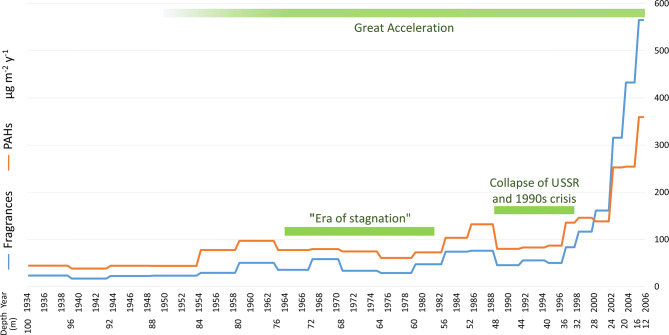
Fluxes of the fragrances and PAHs (µg m^−2^ year^−1^) in the Elbrus ice core. Abscissas report the year of snow deposition (CE) and the sample depth (m).

Similar to the fragrance concentrations, as well as many other indicators of the Great Acceleration^5^, PAHs also increased throughout the studied 70 years. PAH concentrations increased from 49 ng L^−1^ between 1939 and 1944, to a maximum of 166 ng L^−1^ at the top of the core. The corresponding PAH fluxes grew from 38 µg m^−2^ year^−1^ to 359 µg m^−2^ year^−1^ (Fig. 3). The increase in total concentrations of both fragrances and PAHs was statistically significant when comparing the bottom and topmost samples (n = 5 for each), through paired Student’s *t*-tests, with *p* values of *p* = 0.0106 for fragrances and *p* = 0.0016 for PAHs. Light PAHs dominate the PAH profile, with naphthalene (NAP), acenaphthene (ACE) and phenanthrene (PHE) respectively constituting 20 ± 7%, 19 ± 8% and 25 ± 9% of the total PAHs in the samples (Table SI4), followed by fluorene (FLU; 7 ± 1%), fluoranthene (FLA; 8 ± 2%) and pyrene (PYR; 7 ± 2%). The remaining compounds result in less than 3% of the concentrations on average.

The concentrations of the three major PAHs (NAP, ACE and PHE) oscillate throughout the study period (Fig. 2), with relative maxima in the early 1960s and late 1980s, and with their highest peaks in the 2000s. Most of the other PAHs essentially follow a similar scheme. However, two heavy isomers benzo(*ghi*)perylene (B(*ghi*)P) and indeno(1,2,3-*c,d*)pyrene (I(*cd*)P) have a different trend, and rise from the 1960s until 2000, but then rapidly decrease (Fig. 2). Benzo(*b*)fluoranthene B(*b*)F and benzo(*k*)fluoranthene B(*k*)F are found only in the most recent samples.

## Discussion

### Fragrance pollution in the Elbrus ice core

Although personal care products are generally considered to be contaminants with a low environmental mobility^9^, the presence of the fragrances on the summit of the Elbrus icefield can primarily be explained by the cold condensation of long-range transported contaminants, as suggested by the literature^8,28–30^. The concentrations of the fragrances detected in the most recent samples are higher than those of the same compounds found in other remote regions. Total fragrances range from 130 to 281 ng L^−1^ in the uppermost samples of the Elbrus core (Table SI3), while total concentrations remained below 72 ng L^−1^ in Arctic surface snow collected near Ny-Ålesund (Svalbard)^8^, even with the possible local contribution from the scientific bases. Fragrance concentrations in background snow subject to LRAT were up to 10 ng L^−1^, while in Kongsfjorden seawater the fragrance concentrations remained below 5.8 ng L^−1^ ^8^. In Terra Nova Bay, Antarctica, the concentrations of fragrances in seawater increased from a few ng L^−1^ up to 100 ng L^−1^ during the seasonal melt of the sea ice and of its snow cover^28^. These results suggest that long-range transport of fragrances indirectly influence polar regions, while remote areas in the mid latitudes, such as mountain glaciers, have relatively closer sources of fragrances. Emissions from urban areas may lead to high concentrations (up to 10 µg L^−1^) of fragrances in natural waters^29^. Once released into the environment, the fragrances also likely spread to areas far from the emission sources, leading to their deposition and detection in remote regions.

The presence of fragrances in the lowest samples, dated to 1934, is surprising. Benzyl Salicylate was commercialized in the 1930s as one of the first sunscreen agents^33^. As the synthesized volume of this chemical during that decade was probably negligible, an early industrial source is unlikely to explain the detected levels at the bottom of the core. However, traces of Salicylates exist in essential oils extracted from various flowers and plant tissues^32^, thereby constituting a possible natural source of these compounds. The presence of Oranger Crystals in the earliest samples may also be attributed to non-anthropogenic vegetal sources, as this fragrance was detected in corn bud oil extracts^34^. The increase in the fragrance fluxes from the 1950s onward reflects the Great Acceleration in the use and industrial production of the fragrances due to the socioeconomic development in the twentieth century. Assuming that the fragrances in the deepest samples have a natural vegetal origin, it is possible to estimate that their anthropogenic industrial release in the 2000s was 20 times higher than their natural contributions (Fig. 3). Despite remarkable variations, the relative percentages of Benzyl, Amyl, and Hexyl Salicylate remained similar throughout the ice core (Table SI3), implying that the industrial production of the three salicylates as well as their release to the environment increased in parallel. Moreover, despite the fact that urban discharge may emit a wide variety of fragrances^29^, only a few compounds were detected in remote sites, including Elbrus. These limited compounds suggest the possibility of differential transport and/or degradation processes, which should be further investigated.

The flux of Benzyl Salicylate present at the top of the Elbrus core is roughly consistent with its estimated emissions where the worldwide consumption of this compound was 5,700 tons (≈ 5 × 10^15^ µg year^−1^) in 2000^32^. As a proof of concept, by hypothesizing a homogeneous deposition of the yearly production on the Earth surface (≈ 5 × 10^14^ m^2^), we can conservatively estimate an average global flux of 10 µg m^−2^ year^−1^ of Benzyl Salicylate. This value is comparable to that found during the same period on Elbrus (42 µg m^−2^ year^−1^), considering possible preferential condensation phenomena in cold temperatures and that the site is located in a relatively densely populated region of the world, where the emissions are expected to be higher. The salicylate flux increased in the ice layers deposited from 2000 onward, which may reflect the doubling of total global consumption from 2000 to 2010^32^. The faster growth detected in the Elbrus core suggests a more intense increase in the usage of these personal care products in the source regions during the 2000s compared to the global average.

### The evolution of PAHs

The PAH flux increased by almost one order of magnitude during the considered time span, paralleling the Great Acceleration of fragrances. However, the relative growth in PAHs is less pronounced. The fragrance deposition remains approximatively half that of the PAHs for most the investigated period (Fig. 3). The fragrance flux surpasses the PAH deposition at approximately the year 2000, further highlighting the rise of the role of personal care products as an environmental contaminant.

All other studies reporting the distribution of PAHs in ice cores reveal a significant increase in concentrations during the twentieth century. However, regional sources and transport processes may influence specific trends in different areas of the world. For example, PAH concentrations in an Everest ice core gradually increased from 1970 to 1990 and peaked at 100 ng L^−1^ at the end of the 1990s, mainly reflecting the economic and industrial growth of India^15^. Unlike in the Elbrus ice core, the Everest PAH concentrations decrease after 2000 due to changing Indian combustion and energy sources. This decrease in the PAH deposition fluxes after the year 2000 is also detected in another Himalayan firn core from Xixiabangma (Dasuopu), with concentrations below 26 ng L^−1^ ^16^. The PAH history recorded in European ice cores shows different trends. In the Italian Colle Gnifetti ice core, preindustrial PAH concentrations are below 2 ng L^−1^ and begin to increase at the end of nineteenth century, until reaching a maximum concentration of 32 ng L^−1^ in approximatively 1950. After this peak, the concentrations decreased significantly until 1975, probably reflecting improvements in emission controls, yet started to rise again until the top of the core (2003)^17^.

While PAH histories archived in mountain ice cores vary by region, PAHs recorded in Arctic ice tend to have similar trends with one another. The Site-J, Greenland core contains a marked increase in PAHs during the last 400 years where PAH concentrations were generally very low before the eighteenth century (mean 2.3 ng L^−1^) but substantially increased since the 1930s onwards, peaking up to 230 ng L^−1^ at the end of the 1980s^11^. The range in NAP concentrations in the Elbrus core (8–31 ng L^−1^; Table SI4) are similar to those in a Svalbard ice core (5–53 ng L^−1^)^12^, although the trends differ between the cores. The Svalbard NAP concentrations are similar to the total PAH concentrations in Site-J, where the concentrations are below the detection limit prior to the 1930s, peak in the 1980s and then decrease in the following years. The Elbrus NAP concentrations peak in the early 1960s and late 1980s, followed by the largest peak from 2000 onward. Antarctic PAH contamination is generally less than PAH concentrations in Arctic and high mountain areas. At Talos Dome, coastal East Antarctica, PAH concentrations increase from 2.2 ng L^−1^ in 1930 to only 3.2 ng L^−1^ in 2002, where these PAHs are attributed to anthropogenic sources^13^. Such lower levels and fluxes are likely caused by the isolation of the Antarctic continent due to the Antarctic circumpolar current and associated atmospheric influences compared to the Arctic and populated regions, such as the Caucasus. In another Antarctic ice core (GV7, Victoria Land), PAH concentrations slightly increase from background levels less than 5 ng L^−1^ to a nearly constant level of 6.5 ng L^−1^ between 2000 and 2010^14^. However, individual PAH peaks do occur during this time period, with maxima up to 9 ng L^−1^, which correlate with major explosive volcanic eruptions. Sulphate peaks reflecting global scale eruptions, as well as possible local inputs from Elbrus^20,27^, were only found below the ice sections analyzed in this study. Therefore the Elbrus PAH record should be unaffected by relevant volcanic sources.

Compared to the relatively few studies of PAHs in ice cores, more information is available about PAHs in the surface snow of high mountain environments. These PAH concentrations substantially differ by region. The most recent Elbrus PAH concentrations (112–166 ng L^−1^; Table SI4) are comparable to PAHs recorded in Himalayan and Tibetan glaciers. Surface snow concentrations collected in 2011 increase with altitude on Mount Nanshan, Xinjiang, ranging from 70 to 156 ng L^−1^ ^35^. A north to south transect of glaciers conducted in 2008 across the Tibetan Plateau demonstrates mean concentrations in the range of 20–61 ng L^−1^, with the highest concentrations occurring in the central plateau^36^. Snow from Mt. Gongga in Sichuan Province, China collected in 2012–2014 contains PAH concentrations of 290–452 ng L^−1^ which may be due to its relative proximity to urban centers, suggesting coal combustion and traffic emissions as major sources^37^. The continental-scale smog cloud, the “Asian Brown Cloud”, that gathers during the non-monsoon months is a substantial source of PAHs to the southern Himalaya, but it is still unknown if these contaminants are transported northward into the Tibetan Plateau^15^.

Sites in the European Alps generally contain lower concentrations of PAHs compared to Elbrus and the Himalayas. Studies of snow in the Swiss and Austrian Alps in 1997–1998 record respective concentrations of 16 ng L^−1^ and 17 ng L^−1^ ^38^. Later, in 2006, a study of snow in the Tyrolean Alps, Austria, demonstrates a PAH concentration range of 0.5–8.4 ng L^−1^ ^39^. Results from 2005 to 2009 deposition in a shallow firn core from Ortles, located in the eastern Italian Alps, encompass PAH concentrations of 0.5–6.2 ng L^−1^ ^40^. Relatively higher concentrations (20–59 ng L^−1^) were detected in seasonal snowpack sampled in 2004–2005 in the Dolomites (> 1,700 m a.s.l.), with values up to 290 ng L^−1^ in urban areas in valley bottoms^41^. Even lower levels were found in the Cascade Mountains, Oregon, USA, during the winter of 2012–2013, where PAHs were detected in freshly fallen snow at concentrations of 1.3–2.2 ng L^−1^ ^42^. PAH concentrations in snow from the Tatra mountains, Slovakia, are more similar to the levels in the Elbrus core, rising from 81 ng L^−1^ in 1997–1998^38^ to 90–300 ng L^−1^ in 2005^43^. PAHs in surface snow collected in 1992–1993 in the Ob-Yenisey river watershed, arctic Russia, contain extremely high concentrations (≈ 30 µg L^−1^)^44^, while 2016 surface snow from the Novaya Zemlya Archipelago contain FLU and PYR concentrations below 30 ng L^−1^ ^45^. Comparable levels (35–80 ng L^−1^) were also found in snow collected in southern far east Russia^46^. Each of these sampling sites are influenced by their regional sources. Therefore, the Elbrus PAH history helps to close a gap in the knowledge of PAH trends in the Caucasus and in Central/Eastern Europe.

The elevation gradient of mountains impacts the PAH distribution in high-altitude environments, including Elbrus. Heavy compounds are preferentially deposited at lower altitudes, while light compounds such as PHE are more prevalent at high elevations, resulting in a higher percentage of light PAHs in the Elbrus ice core^16,38^. The majority of ice core^11,13,15,17^ and surface snow^16,35–37,43^ studies indicate that combustion processes are the major sources of PAHs. Diagnostic molecular ratios are widely used to discriminate the sources of PAHs^47^. The FLA/(FLA + PYR) ratio is consistently > 0.5 throughout the Elbrus core (Table SI4), indicating biomass and coal combustion. However, the ANT/(ANT + PHE) (where ANT = anthracene) ratio is always < 0.1 suggesting petrogenic sources. This apparent contradiction in the diagnostic ratios may be influenced by the differing characteristic travel distances of the PAHs during atmospheric transport, where isomers may have differing reactivities^48^. LRAT influences these ratios where FLA/(FLA + PYR) tends to increase with distance, while ANT/(ANT + PHE) decreases. This transport effect can result in possibly assuming an inaccurate source, regardless of the original emissions (pyrogenic *vs.* petrogenic). The Elbrus data may be affected by this ageing process of the diagnostic ratios^48^. Similar weathering processes occur during LRAT for benzo(*a*)anthracene (B(*a*)A) and chrysene (CHR), resulting in the more stable ratio B(*a*)A/(B(*a*)A + CHR) ratio that, when applicable in the Elbrus ice core, is usually < 0.2 indicating petrogenic sources (Table SI4). The I(*cd*)P/(I(*cd*)P + B(*ghi*)P) ratio remains relatively stable in the transfer from the atmosphere to the other compartments, where results > 0.5 suggest biomass and coal combustion (Table SI4). Considering these limitations, diagnostic ratios should be used with caution in remote high-altitudes sites.

Petrogenic sources may in principle influence the chemical composition of snow on Elbrus through dust deposition originating from oil producing countries in the Middle East and North Africa^23,24^. This input is a distinctive characteristic of Elbrus, and differs from other high-altitude PAH records, due to the geographical position of the Caucasus region. Other considerations lean toward a predominance of pyrogenic sources: Retene (RET; 1-methyl-7-isopropyl phenanthrene) can naturally derive from the degradation of abietic acid and may be present in some types of coal, yet is also a typical tracer of coniferous wood combustion, and is released during forest fires^8,19^. The increasing trend in RET throughout the Elbrus core (Table SI4), follows the general PAH profile, and adds support to the diagnostic ratios, suggesting combustion as a relevant source of PAHs.

PAHs in ice cores from Svalbard, the Himalayas, and the Italian Alps decrease in recent years where this decline is attributed to improvements in pollution reduction policies^12,15,17^. B(*ghi*)P and I(*cd*)P are generally considered to be tracers of industrial processes and gasoline vehicular emissions^18^, where their decline in the mid-2000s may be linked to improvements in emission controls. However, concentrations of other heavy PAHs continue to increase (Table SI4). Despite a reduction in the estimated emissions in Europe from 1990 to 2005^18^, benzo(*a*)pyrene (B(*a*)P) in the Elbrus ice core increased from 1–1.5 to 4 ng L^−1^. A similar phenomenon was detected in Arctic air, where B(*a*)P concentrations did not decline^49^. In the Elbrus core, benzo(*b*)fluoranthene (B(*b*)F) and benzo(*k*)fluoranthene (B(*k*)F) only had concentrations comparable to or higher than B(*a*)P after the year 2000 (Table SI4). In Himalayan, Tibetan and Xinjiang sites^15,35–37^, as well as in Japanese snow^50^ B(*b*)F and B(*k*)F concentrations consistently remain higher than B(*a*)P. European snow samples usually also show the same prevalence of B(*b*)F and B(*k*)F^17,38–41,43^. In Arctic sites this predominance is generally less pronounced: the three isomers exist at similar levels in Svalbard snow^8,51^, while B(*a*)P prevailed in northwestern Canadian locations and far east Russian background sites^46,52^. In Antarctica, a clear B(*a*)P predominance exists in Northern Victoria Land^53^ and in the Talos Dome ice core^13^. The coastal Antarctic site GV7 is influenced by different deposition regimes^14^, resulting in higher concentrations of B(*k*)F. These differences indicate that the relative abundance of B(*a*)P, B(*b*)F and B(*k*)F in snow may reflect the influence of the regional sources. Consequently, the concentrations of B(*a*)P, B(*b*)F and B(*k*)F suggest a concurrent shift in the emission patterns of heavy PAHs occurred in the source regions of air masses that reach the Elbrus icefield^21,22^.

A Cluster Analysis of the relative distribution of the compounds (Figure SI1) also demonstrates PAH variations in the Elbrus ice core. Four different clusters were identified, which correspond to different time periods within the core. Relevant exceptions to these time periods include the PAH concentration maxima in the 1960s and 1980s (samples at 48–52 m and 76–80 m depth), which are clustered together with the more contaminated samples deriving from the top of the core. The samples that correspond to the economic crisis of the 1990s (32–36 m and 36–40 m depth) are clustered with the 1970s samples, thereby confirming the peculiarity of their composition.

### Acceleration and crises

**Figure 4:**
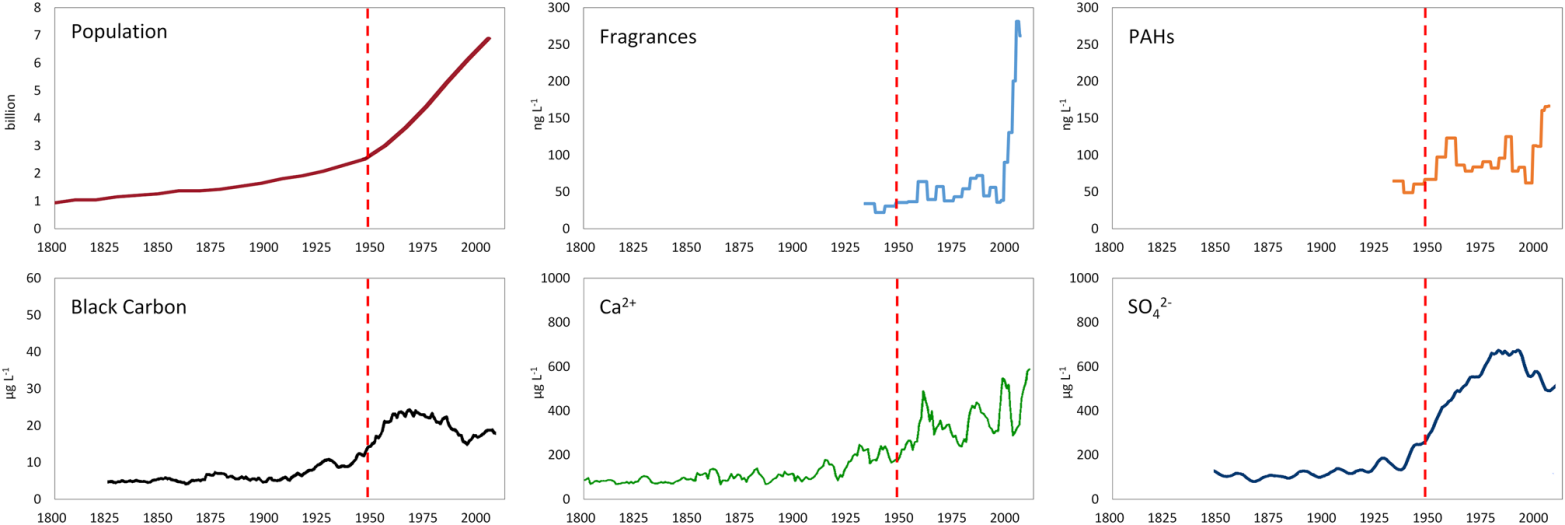
Multiple signals of the Great Acceleration archived in the Elbrus ice core compared to the trend of the global population as reported by Steffen et al.^54^. Concentrations of fragrances and PAHs (ng L^−1^) are from this study. The 5-year moving averages of the summer concentrations (µg L^−1^) of Black Carbon (BC), Ca^2+^ as a proxy for dust, and sulphate (SO_4_^2−^) were published elsewhere^22,25,27^.

The general increasing trend of personal care products and PAHs detected in the second half of the twentieth century in the Elbrus core is remarkable. This evolution also agrees with the trends of BC^22^, sulphate^27^ and dust^25^ analyzed in the Elbrus core (Fig. 4). The contemporaneous acceleration of different and independent proxies around 1950 is one of the main features of the Anthropocene^54^, revealing the impact of the human imprint across the globe including in high-altitude environments.

The combustion tracers, PAHs and BC, show an overall similar trend throughout the Elbrus ice core^22^. BC concentrations are clearly higher in summer than in winter deposition, with a 1.5-fold increase in the first half of the twentieth century over its preindustrial level. Annual mean BC concentrations increased from 1940, peaked in 1980 and afterwards declined until the year 2000, when they started to rise again (Fig. 4). This recent increase was even more pronounced for PAHs (Fig. 3), which in the mid-2000s surpassed the concentrations of the 1960s–1980s. An individual maximum BC mass concentration of 222 µg L^−1^ occurred during in the summer of 2003, corresponding to extreme forest fire events in Southern Europe^22^. Sulphate deposition in the Elbrus core follows a similar trend with a maximum between 1980 and 1990, with higher concentrations during each summer^27^. This trend is consistent with increased coal combustion from Central Europe and the Levant rather than from Western Europe. Summer Ca^2+^ concentrations, a proxy for dust deposition, increased from the mid-twentieth century onward^25^, reflecting more frequent droughts in North Africa and the Middle East, due to a warming climate and anthropogenic land use change (Fig. 4).

Although the fragrance and PAH concentrations in the Elbrus ice core increase from the 1950s onward, their trends include two major decreases during this time period (Fig. 3). These reductions in the analyte fluxes are synchronous with socioeconomic crises that occurred in Central and Eastern Europe. Mikhail Gorbachev defined the period between 1964 and 1982, when the USSR was ruled by his predecessor Leonid Brezhnev, as the “era of stagnation”. The economy was not entirely stagnant during this time period but rather only experienced slow economic growth^55^. From 1945 onward, the real per capita GDP in the USSR grew almost continuously, yet at different rates, and declined only in 1963 and 1979 due to severe harvest failures^55^. The samples encompassing these years showed relative deposition minima for both fragrances and PAHs (Fig. 3). The second decrease in analyte concentrations corresponds to the crisis of the 1990s. The communist system collapsed between 1989 and 1991 with catastrophic consequences for the population in the following years, resulting in an unprecedented health crisis: the life expectancy in Russia dropped from 70.13 years in 1986–87 to 65.93 years in 1999, with a minimum of 63.96 years in 1994^56^. By the end of 1995, over 35% of the Russian population was living below the official poverty line^57^. The associated changes in food consumption, especially beef, and the vegetation recolonization of abandoned cropland resulted in a net cumulative reduction of carbon dioxide emissions^58^. The financial crisis resulted in the collapse of the ruble in August 1998, even though other European ex-communist republics experienced substantial economic growth during the same time period^56^. Mirroring the hardest years of the crisis, concentrations of both fragrances and PAHs dropped after 1989, and the two samples encompassing the period between the end of 1994 and the end of 1997 contain the lowest concentrations of any time since the 1940s (Fig. 2). However, the relatively higher flux estimates in the latter sample may reflect more intense snow deposition (Fig. 3). The ex-USSR and associated countries are the closest sources for fragrances and PAHs (Figure SI1), but are not the only source of atmospheric aerosols that influence Elbrus^21,22^. Nevertheless, the trends in the fragrance and PAH fluxes in the Elbrus ice core reflect the major socioeconomic crises occurring in Eastern Europe during the twentieth century, overlain on the growth in anthropogenic chemicals of the Great Acceleration.

## Methods

The sampling site and the drilling operations were previously described in detail in Mikhalenko et al.^20^. Briefly, a 181.8 m ice core was drilled to bedrock in September 2009 and shipped in a frozen state to the Lomonosov Moscow State University for preliminary investigation. The core was analyzed for stable isotopes using discrete samples at the Arctic and Antarctic Research Institute in St. Petersburg, Russia^21^. The core was also sampled using a Continuous Flow Analysis (CFA) system (ice core sticks; 3.4 cm × 3.4 cm × 1 m) at the Institut des Géosciences de l’Environnement (IGE) in Grenoble, France. CFA streams were routed for dust, major ions, and black carbon analyses. The final CFA stream was collected in 1 L glass jars precleaned with pesticide-grade solvents and refrozen. Samples were later melted in the stainless steel clean-room laboratories for organic analyses (class 10,000) of the Ca’ Foscari University of Venice, Italy, and extracted using 200 mg Oasis^®^ HLB cartridges (Waters) following previously developed methods^8,29^. In order to obtain appropriate volumes for extractions (0.316–1.560 L), we combined four consecutive samples. This combination resulted in 22 samples, corresponding to the depth interval from 12 to 100 m. Combining the samples resulted in an annual resolution at the top of the core, and a resolution of approximately 5 years per sample at the bottom. Fluxes were calculated as concentrations in ice multiplied for the height of water equivalents and divided by the years of deposition, using a 0.5 years resolution as determined by seasonal layers. Multiple and independent techniques were used to date the core, including annual layer counting, stable isotopes and major ions analyses, cross-checked with radioisotopes and volcanic spikes. Dating details of the core are available in Mikhalenko et al.^20^ and Kozachek et al.^21^.

The relevant physico-chemical properties of the selected fragrances (Givaudan^®^, Vernier, Switzerland; Amberketal, Ambrofix, Amyl Salicylate, Benzyl Salicylate, Bourgeonal, Dupical, Hexyl Salicylate, Isobutavan, Lemonile, Mefranal, Myraldene, Okoumal, Oranger Crystals, Pelargene, Peonile, Tridecene-2-Nitrile, Ultravanil) are described elsewhere^29,30^. The standard solutions for native PAHs (Naphtalene (NAP), acenaphthylene (ACY), acenaphthene (ACE), fluorene (FLU), phenanthrene (PHE), anthracene (ANT), fluoranthene (FLA), pyrene (PYR), benzo(*a*)anthracene (B(*a*)A), chrysene (CHR), retene (RET), benzo(*b*)fluoranthene (B(*b*)F), benzo(*k*)fluoranthene (B(*k*)F), benzo(*a*)pyrene (B(*a*)P), benzo(*ghi*)perylene (B(*ghi*)P), indeno(1,2,3-*c,d*)pyrene (I(*cd*)P) and dibenzo(*a,h*)anthracene (D(*a,h*)A)) were acquired from Dr. Ehrenstorfer (Augsburg, Germany). Isotope-labelled standard solutions (Cambridge Isotope Laboratories; CLM-2477, CLM-2451, CLM-2722) were used as internal standards. Cartridges were eluted with 1 mL of toluene, 15 mL of dichloromethane and 10 mL of *n*-hexane (pesticide-grade, Romil Ltd) and eluates were concentrated under a gentle nitrogen flow at 23 °C (Turbovap II^®^, Caliper Life Science). Instrumental analyses were conducted in Single Ion Monitoring by GC–MS (7890A-5975C, Agilent Technologies) on a 60-m HP-5MS column (0.25 mm I.D., 0.25 μm; Agilent Technologies). Results were corrected using the instrumental response factors and procedural blanks (n = 3) conducted among samples extracting ultrapure water. The mean method recoveries were 94 ± 6% for PAHs and 84 ± 5% for fragrances, while trueness resulted on average respectively the 99 ± 4% and 88 ± 12% of the spiked values. The method detection limit was calculated as three times the standard deviation of the blank signal (Table SI2). Fragrance concentrations below method detection limits in any sample (Amberketal, Bourgeonal, Dupical, Isobutavan, Lemonile, Mefranal, Myraldene, Pelargene, Tridecene-2-Nitrile, Ultravanil) were excluded from the discussion.

## Supplementary information

Supplementary information
