# Supplementary material for: The Great Acceleration of fragrances and PAHs archived in an ice core from Elbrus, Caucasus
Source: Sci Rep. 2020 Jun 30;10:10661. doi: 10.1038/s41598-020-67642-x (PMC7327048; doi:10.1038/s41598-020-67642-x)
Supplement: Supplementary file 1 — Supplementary information [file 41598_2020_67642_MOESM1_ESM.doc]

**The Great Acceleration of Fragrances and PAHs archived in an ice core from Elbrus, Caucasus. Supplementary material**

Marco Vecchiato, Andrea Gambaro, Natalie M. Kehrwald, Patrick Ginot, Stanislav Kutuzov, Vladimir Mikhalenko, Carlo Barbante.

**Table SI1**: Name, CAS and formulas of the detected fragrances (Givaudan®). The molecular weight (MW), Boiling Point (BP °C at 1 atm) vapour pressure at 25°C (Pa), octanol-water partition coefficient (Log P_ow_) and Water Solubility (mg/L at 20°C) and are also displayed (European Chemicals Agency (ECHA): echa.europa.eu).

| **Name** | | **CAS#** | **Formula** | **MW** | **BP** | **Pa** | **LogPow** | **W. Sol.** |
| --- | --- | --- | --- | --- | --- | --- | --- | --- |
| **Amyl Salicylate** | Pentyl 2-hydroxybenzoate & 3-Methylbutyl 2-hydroxybenzoate | 2050-08-0 87-20-7 | C12H16O3  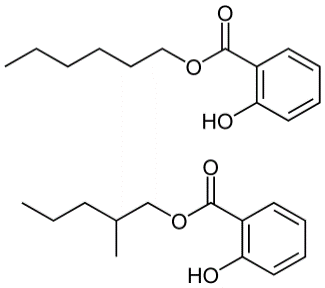 | 208 | 282 | 0.24 | 4.4 | 5.5 |
| **Oranger Crystals** | 2-Acetonaphthone | 93-08-3 | C12H10O  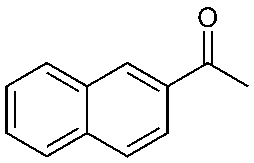 | 170 | 300 | 0.12 | 2.678 | 133.3 |
| **Hexyl Salicylate** | Hexyl 2-hydroxybenzoate | 6259-76-3 | C13H18O3  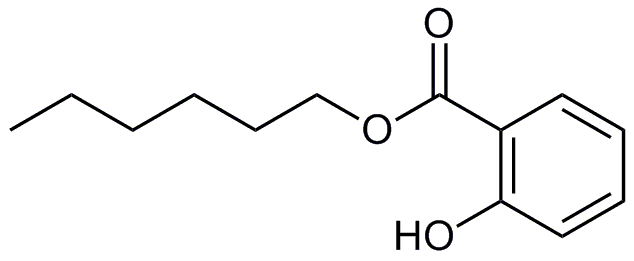 | 222 | 298 | 0.077 | 5.5 | 2 |
| **Ambrofix / Ambroxide** | Dodecahydro-3a,6,6,9a-tetramethylnaphtho[2,1-b]furan | 6790-58-5 | C16H28O  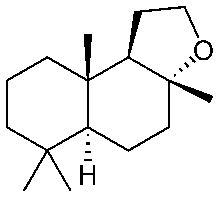 | 236 | 318 | 0.066 | 5.09 | 1.88 |
| **Peonile** | 2-Cyclohexylidene-2-phenylacetonitrile | 10461-98-0 | C14H15N  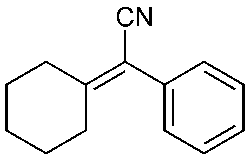 | 197 | 250 | 0.043 | 2 | 7.5 |
| **Okoumal** | 2,4-Dimethyl-2-(1,1,4,4-tetramethyltetralin-6-yl)-1,3-dioxolane (trans & cis isomers) | 131812-67-4 / 131812-51-6 / 131812-52-7 | C19H28O2  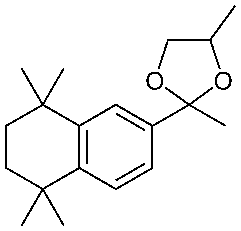 | 288 | 303 | 0.06 | 5.7 | 8 |
| **Benzyl Salicylate** | Benzyl 2-hydroxybenzoate | 118-58-1 | C14H12O3  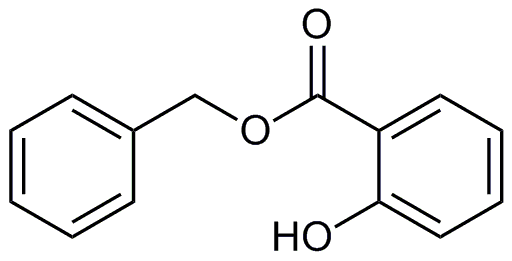 | 228 | 322 | 0.01 | 4 | 8.8 |


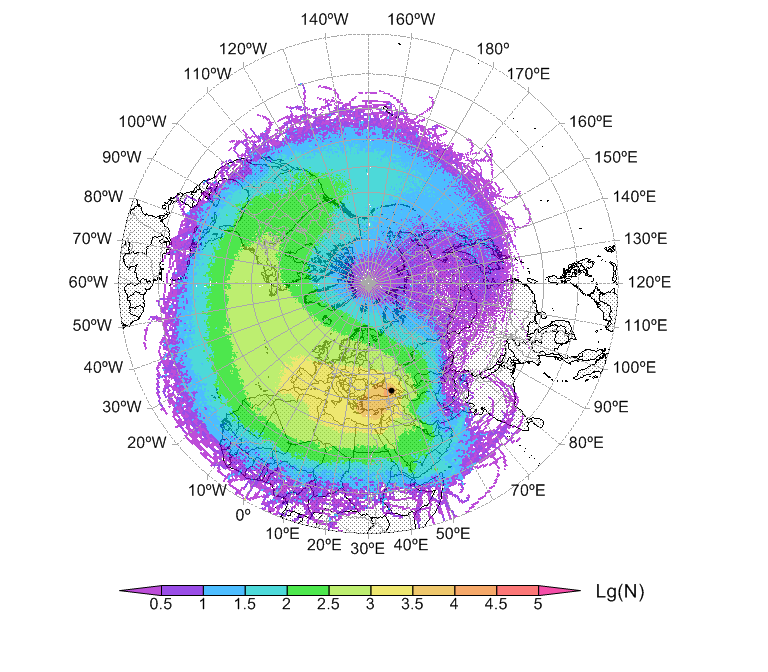


**Figure SI1**: Density plot of 10-day back-trajectories starting from the drilling point and computed every 6 h using the NOAA-HYSPLIT model and NCEP/NCAR Reanalysis database for the period 1948-2013. The number of trajectories (N) is reported in logarithmic scale.

**Table SI2**: Ions (m/z) selected for quantification and identification of the analytes. Mean blanks, Method Detection Limits (MLD) and Instrumental Detection Limits (IDL). MDL was calculated as three times the standard deviation of the blank signal and the results reported in the table SI3 are blank corrected.

| **Fragrances** | Quantifier Ion | Qualifier Ion | Mean blank (ng L^-1^) | | | MDL (ng L^-1^) | | IDL (pg) | |
| --- | --- | --- | --- | --- | --- | --- | --- | --- | --- |
| Ultravanil | 124.1 | 152.1 | - | - | | - | | 18 | |
| Lemonile^a^ | 148.1 | 162.1 | - | - | | - | | 83 | |
| Pelargene | 129.1 | 188.1 | - | - | | - | | 3 | |
| Mefranal | 91.1 | 176.1 | - | - | | - | | 5 | |
| Bourgeonal^b^ | 175.1 | 190.1 | 3.1 ± | 2.3 | | 6.9 | | 4 | |
| Myraldene^b^ | 192.1 | 149.1 | - | - | | - | | 18 | |
| Tridecene-2-nitrile^b^ | 164.1 | 192.1 | - | - | | - | | 3 | |
| Amyl Salicylate^b^ | 120.1 | 208.1 | 6.9 ± | 2.3 | | 7.0 | | 4 | |
| Oranger Crystals | 155.1 | 170.1 | 0.3 ± | 0.1 | | 0.3 | | 35 | |
| Dupical^b^ | 135.1 | 120.1 | - | - | | - | | 38 | |
| Isobutavan | 152.1 | 222.1 | 0.6 ± | 0.1 | | 0.2 | | 3 | |
| Hexyl Salicylate | 120.1 | 222.1 | 2.5 ± | 0.3 | | 1.0 | | 4 | |
| Ambrofix | 221.1 | 236.4 | 1.4 ± | 0.4 | | 1.3 | | 3 | |
| Peonile | 197.1 | 130.1 | 2.8 ± | 1.1 | | 3.3 | | 3 | |
| Okoumal | 273.1 | 215.2 | 0.2 ± | 0.2 | | 0.6 | | 4 | |
| Benzyl Salicylate | 91.1 | 228.1 | 0.5 ± | 0.2 | | 0.7 | | 5 | |
| Amberketal | 190.2 | 218.2 | - | - | | - | | 18 | |
| **PAHs** | Quantifier and Qualifier Ion | | Mean blank (ng L^-1^) | | | | MDL (ng L^-1^) | | IDL (pg) |
| NAP | 128.1 | | 10.5 ± | | 0.2 | | 0.5 | | 0.001 |
| ACY | 152.1 | | 0.2 ± | | 0.1 | | 0.2 | | 0.001 |
| ACE | 154.1 | | 10.2 ± | | 1.2 | | 3.5 | | 0.01 |
| FLU | 166.1 | | 0.6 ± | | 0.2 | | 0.5 | | 0.01 |
| PHE | 178.1 | | 1.5 ± | | 0.3 | | 0.9 | | 0.01 |
| ANT | 178.1 | | 0.04 ± | | 0.01 | | 0.04 | | 0.01 |
| FLA | 202.1 | | - | | - | | - | | 0.01 |
| PYR | 202.1 | | - | | - | | - | | 0.01 |
| B(*a*)A | 228.1 | | - | | - | | - | | 1 |
| CHR | 228.1 | | 0.04 ± | | 0.02 | | 0.1 | | 1 |
| RET | 234.1 | | 0.1 ± | | 0.1 | | 0.4 | | 2 |
| B(*b*)F | 252.1 | | 0.2 ± | | 0.1 | | 0.2 | | 1 |
| B(*k*)F | 252.1 | | 0.1 ± | | 0.004 | | 0.01 | | 1 |
| B(*a*)P | 252.1 | | 1.8 ± | | 0.1 | | 0.2 | | 1 |
| B(*ghi*)P | 276.1 | | 0.03 ± | | 0.01 | | 0.02 | | 2 |
| I(*cd*)P | 276.1 | | 0.4 ± | | 0.3 | | 0.9 | | 2 |
| D(*ah*)A | 278.1 | | 0.03 ± | | 0.003 | | 0.01 | | 2 |

^a^ Sum of four isomers. ^b^ Sum of two isomers.

**Table SI3**: Concentrations of fragrances (ng L^-1^) in the Elbrus ice core. (nd: not detected; ldl: lower detection limits)

| Depth (m) | **12-16** | **16-20** | **20-24** | **24-28** | **28-32** | **32-36** | **36-40** | **40-44** | **44-48** | **48-52** | **52-56** | **56-60** | **60-64** | **64-68** | **68-72** | **72-76** | **76-80** | **80-84** | **84-88** | **88-92** | **92-96** | **96-100** |
| --- | --- | --- | --- | --- | --- | --- | --- | --- | --- | --- | --- | --- | --- | --- | --- | --- | --- | --- | --- | --- | --- | --- |
| Ultravanil | nd | nd | nd | nd | nd | nd | nd | nd | nd | nd | nd | nd | nd | nd | nd | nd | nd | nd | nd | nd | nd | nd |
| Lemonile^a^ | nd | nd | nd | nd | nd | nd | nd | nd | nd | nd | nd | nd | nd | nd | nd | nd | nd | nd | nd | nd | nd | nd |
| Pelargene | nd | nd | nd | nd | nd | nd | nd | nd | nd | nd | nd | nd | nd | nd | nd | nd | nd | nd | nd | nd | nd | nd |
| Mefranal | nd | nd | nd | nd | nd | nd | nd | nd | nd | nd | nd | nd | nd | nd | nd | nd | nd | nd | nd | nd | nd | nd |
| Bourgeonal^b^ | ldl | ldl | ldl | ldl | ldl | nd | nd | ldl | nd | nd | ldl | ldl | nd | ldl | ldl | ldl | ldl | ldl | ldl | ldl | ldl | ldl |
| Myraldene^b^ | nd | nd | nd | nd | nd | nd | nd | nd | nd | nd | nd | nd | nd | nd | nd | nd | nd | nd | nd | nd | nd | nd |
| Tridecene-2-nitrile^b^ | nd | nd | nd | nd | nd | nd | nd | nd | nd | nd | nd | nd | nd | nd | nd | nd | nd | nd | nd | nd | nd | nd |
| Amyl Salicylate^b^ | 36 | 65 | 48 | 34 | 25 | 9.1 | 10 | 19 | 13 | 17 | 25 | 16 | 12 | 7.2 | 13 | 9.4 | 10 | 7.6 | 8.6 | 7.9 | 6.2 | 7.6 |
| Oranger Crystals | 10 | 10 | 6.4 | 6.1 | 3.4 | 3.0 | 1.2 | 1.1 | 1.7 | 3.9 | 6.8 | 8.2 | 4.8 | 4.1 | 4.4 | 3.7 | 5.3 | 2.4 | 2.1 | 1.2 | 0.8 | 2.2 |
| Dupical^b^ | nd | nd | nd | nd | nd | nd | nd | nd | nd | nd | nd | nd | nd | nd | nd | nd | nd | nd | nd | nd | nd | nd |
| Isobutavan | ldl | ldl | ldl | ldl | ldl | ldl | nd | nd | ldl | ldl | ldl | ldl | ldl | ldl | ldl | ldl | ldl | nd | ldl | ldl | ldl | ldl |
| Hexyl Salicylate | 85 | 102 | 67 | 44 | 31 | 12 | 13 | 22 | 14 | 31 | 17 | 13 | 13 | 11 | 18 | 13 | 24 | 14 | 14 | 13 | 7.2 | 12 |
| Ambrofix | 10 | 7.1 | 4.2 | 3.3 | ldl | 1.3 | ldl | 1.2 | ldl | 1.9 | 3.1 | 2.5 | ldl | 2.0 | 2.1 | ldl | ldl | ldl | ldl | ldl | ldl | ldl |
| Peonile | 13 | 15 | 14 | 9.1 | 6.0 | 3.4 | 3.7 | ldl | 5.2 | ldl | 3.6 | ldl | ldl | ldl | ldl | ldl | ldl | ldl | ldl | ldl | ldl | ldl |
| Okoumal | 10 | ldl | ldl | ldl | ldl | ldl | ldl | ldl | ldl | ldl | ldl | ldl | ldl | ldl | 1.7 | ldl | ldl | ldl | ldl | ldl | ldl | ldl |
| Benzyl Salicylate | 98 | 82 | 61 | 34 | 24 | 9.4 | 7.9 | 13 | 10 | 18 | 13 | 15 | 13 | 14 | 18 | 14 | 24 | 13 | 11 | 9.1 | 7.9 | 12 |
| Amberketal | nd | nd | nd | nd | nd | nd | nd | nd | nd | nd | nd | nd | nd | nd | nd | nd | nd | nd | nd | nd | nd | nd |
| **Total FMs (ng L^-1^)** | 262 | 281 | 200 | 130 | 90 | 38 | 36 | 56 | 45 | 72 | 68 | 54 | 43 | 38 | 57 | 40 | 64 | 37 | 35 | 31 | 22 | 34 |

^a^ Sum of four isomers. ^b^ Sum of two isomers.

**Table SI4**: Concentrations of PAHs (ng L^-1^) in the Elbrus ice core. (nd: not detected; ldl: lower detection limits). PAHs Diagnostic Ratios (Tobiszewski and Namieśnik. 2012) are reported (na: not applicable).

| Depth (m) | **12-16** | **16-20** | **20-24** | **24-28** | **28-32** | **32-36** | **36-40** | **40-44** | **44-48** | **48-52** | **52-56** | **56-60** | **60-64** | **64-68** | **68-72** | **72-76** | **76-80** | **80-84** | **84-88** | **88-92** | **92-96** | **96-100** |
| --- | --- | --- | --- | --- | --- | --- | --- | --- | --- | --- | --- | --- | --- | --- | --- | --- | --- | --- | --- | --- | --- | --- |
| NAP | 27 | 20 | 15 | 24 | 31 | 12 | 15 | 24 | 16 | 9.9 | 19 | 13 | 15 | 7.9 | 13 | 19 | 21 | 26 | 16 | 16 | 15 | 21 |
| ACY | 2.1 | ldl | ldl | 1.3 | 0.9 | 0.6 | 0.4 | 0.3 | 0.7 | 1.4 | 1.1 | 0.6 | 1.1 | 0.9 | 1.2 | 1.0 | 0.9 | 2.5 | 0.8 | 1.0 | 0.8 | 2.6 |
| ACE | 17 | 47 | 48 | 32 | 20 | 17 | 16 | 13 | 26 | 17 | 20 | 15 | 18 | 7.4 | 5.9 | 18 | 15 | 18 | 8.1 | 6.9 | 7.3 | 7.5 |
| FLU | 12 | 10 | 8 | 9.6 | 6.4 | 3.7 | 4.0 | 6.0 | 5.8 | 12 | 6.3 | 5.6 | 7.6 | 4.8 | 5.6 | 5.7 | 10 | 6.0 | 3.8 | 3.7 | 2.7 | 5.2 |
| PHE | 67 | 61 | 46 | 23 | 32 | 8.7 | 7.5 | 19 | 11 | 57 | 17 | 20 | 18 | 19 | 21 | 17 | 45 | 20 | 18 | 16 | 9.3 | 13 |
| ANT | 2.0 | nd | 1.6 | 0.7 | 1.2 | 0.7 | 0.6 | 0.6 | 0.6 | 1.5 | 0.7 | 0.5 | 1.0 | 0.7 | 0.9 | 0.6 | nd | 0.5 | 0.4 | 0.3 | 0.2 | 0.5 |
| FLA | 13 | 10 | 13 | 6.9 | 7.1 | 4.4 | 4.0 | 5.3 | 4.8 | 9.2 | 9.2 | 7.2 | 8.8 | 11 | 9.1 | 7.3 | 11 | 7.2 | 5.8 | 5.4 | 4.2 | 4.5 |
| PYR | 9.2 | 5.3 | 9.2 | 5.3 | 6.5 | 4.4 | 3.8 | 4.1 | 3.8 | 7.5 | 8.4 | 6.0 | 7.7 | 10 | 7.3 | 5.5 | 8.1 | 5.8 | 4.9 | 3.9 | 3.2 | 3.4 |
| B(*a*)A | nd | nd | 0.7 | nd | nd | 0.3 | 0.4 | nd | nd | 0.3 | nd | nd | nd | 1.0 | nd | 0.6 | nd | 0.6 | 0.5 | 0.3 | 0.2 | 0.3 |
| CHR | 2.8 | 0.9 | 3.0 | 1.6 | 0.5 | 1.7 | 1.8 | 1.7 | 2.5 | 1.7 | 2.8 | 2.7 | 2.8 | 3.9 | 3.2 | 2.5 | 3.0 | 2.8 | 2.4 | 1.8 | 1.7 | 1.4 |
| RET | 3.2 | nd | 2.3 | 1.4 | nd | 0.9 | 1.2 | 1.0 | 1.0 | 1.4 | 1.0 | 1.3 | 1.3 | 1.8 | 1.0 | 0.7 | 1.6 | 1.2 | 0.6 | 0.6 | 0.4 | 0.9 |
| B(*b*)F | 0.6 | 6.2 | 1.0 | 0.9 | nd | nd | nd | nd | nd | nd | nd | nd | nd | nd | nd | nd | nd | nd | nd | nd | nd | nd |
| B(*k*)F | 5.5 | 0.9 | 3.8 | 0.9 | nd | nd | nd | nd | nd | nd | nd | nd | nd | nd | nd | nd | nd | nd | nd | nd | nd | nd |
| B(*a*)P | 4.0 | 2.8 | 4.1 | 1.7 | 1.6 | 2.2 | 2.1 | 1.0 | 1.5 | 1.5 | 2.7 | 3.3 | 2.5 | 3.7 | 2.5 | 3.0 | 2.1 | 2.3 | 1.8 | 1.5 | 1.6 | 1.8 |
| B(*ghi*)P | 0.3 | 0.2 | 2.5 | 1.5 | 2.1 | 1.9 | 2.0 | 1.9 | 1.7 | 1.8 | 3.7 | 2.8 | 2.7 | 4.0 | 2.7 | 2.2 | 2.2 | 2.4 | 1.9 | 1.2 | 1.2 | 0.9 |
| I(*cd*)P | nd | nd | 2.6 | 1.8 | 3.3 | 3.3 | 2.5 | 5.1 | 2.0 | 2.7 | 3.9 | 4.8 | 4.6 | 6.9 | 3.6 | 2.8 | 2.2 | 2.1 | 2.1 | 1.3 | 1.2 | 1.0 |
| D(*ah*)A | 0.5 | 0.5 | 0.2 | 0.2 | 0.2 | 0.3 | 0.3 | 0.2 | 0.2 | 0.2 | 0.5 | 0.3 | 0.4 | 0.5 | 0.5 | 0.5 | 0.3 | 0.2 | 0.1 | 0.1 | 0.1 | 0.1 |
| **Total PAHs (ng L^-1^)** | **166** | **165** | **160** | **112** | **113** | **62** | **62** | **83** | **78** | **125** | **95** | **82** | **91** | **84** | **78** | **86** | **123** | **97** | **67** | **60** | **49** | **64** |
| ANT/(ANT+PHE) | 0.03 | na | 0.03 | 0.03 | 0.04 | 0.07 | 0.08 | 0.03 | 0.05 | 0.03 | 0.04 | 0.03 | 0.05 | 0.03 | 0.04 | 0.03 | na | 0.02 | 0.02 | 0.02 | 0.02 | 0.04 |
| FLA/(FLA+PYR) | 0.59 | 0.66 | 0.58 | 0.56 | 0.52 | 0.50 | 0.52 | 0.56 | 0.56 | 0.55 | 0.52 | 0.55 | 0.53 | 0.52 | 0.56 | 0.57 | 0.57 | 0.55 | 0.54 | 0.58 | 0.57 | 0.57 |
| B(*a*)A/(B(*a*)A+CHR) | na | na | 0.18 | na | na | 0.15 | 0.17 | na | na | 0.15 | na | na | na | 0.20 | na | 0.19 | na | 0.19 | 0.17 | 0.13 | 0.13 | 0.17 |
| I(*cd*)P/(I(*cd*)P+B(*ghi*)P) | na | na | 0.51 | 0.55 | 0.61 | 0.64 | 0.56 | 0.73 | 0.55 | 0.60 | 0.52 | 0.64 | 0.63 | 0.63 | 0.57 | 0.55 | 0.50 | 0.47 | 0.53 | 0.51 | 0.50 | 0.54 |

**Figure SI2**: Cluster analysis of the distribution of the PAH compounds in the Elbrus ice core. Ward clustering method and Pearson correlation distance type were used on normalized variables (OriginLab). Four different clusters were identified and are highlighted with blue rectangles. These clusters correspond with different sections of the core, while red circles respectively indicate the maxima of the PAH concentrations of 1960s and 1980s and the minima of the 1990s.
